# Phylogenetic relationships of the glycoprotein gene of bovine ephemeral fever virus isolated from mainland China, Taiwan, Japan, Turkey, Israel and Australia

**DOI:** 10.1186/1743-422X-9-268

**Published:** 2012-11-14

**Authors:** Fuying Zheng, Changqing Qiu

**Affiliations:** 1 State Key Laboratory of Veterinary Etiological Biology, Lanzhou Veterinary Research Institute, Chinese Academy of Agricultural Sciences, No. 1 Xujiaping, Yanchangbao, Lanzhou, 730046, China

**Keywords:** Bovine ephemeral fever virus, Glycoprotein gene, Mainland China, Phylogenetic relationship, Variation

## Abstract

**Background:**

The glycoprotein (G) gene sequences of bovine ephemeral fever virus (BEFV) strains derived from mainland China have not been compared with those of the isolates from other countries or areas. Therefore, the G genes of four BEFV isolates obtained from mainland China were amplified and sequenced. A phylogenetic tree was constructed in order to compare and analyze the genetic relationships of the BEFV isolates derived from mainland China and different countries and areas.

**Results:**

The complete BEFV G gene was successfully amplified and sequenced from four isolates that originated from mainland China. A total of fifty-one BEFV strains were analyzed based on the G gene sequence and were found to be highly conserved. A phylogenetic tree showed that the isolates were grouped into three distinct lineages depending on their source of origin. The antigenic sites of G_1_, G_2_ and G_3_ are conserved among the isolates, except for several substitutions in a few strains.

**Conclusions:**

The phylogenetic relationships of the BEFV isolates that originated from mainland China, Taiwan, Japan, Turkey, Israel and Australia were closely related to their source of origin, while the antigenic sites G_1_, G_2_ and G_3_ are conserved among the BEFV isolates used in this work.

## Background

Bovine ephemeral fever virus (BEFV) is an arthropod-borne rhabdovirus which belongs to the genus *Ephemerovirus* in the *Rhabdoviridae*[1]. Bovine ephemeral fever (BEF), caused by BEFV, is an acute febrile disease in cattle and water buffalo in tropical and subtropical regions of Africa, Asia, Australia and the Middle East. The disease has a considerable economic impact on dairy farming in China. Most infected livestock present with a decrease in the quantity and quality of milk, and lameness or paralysis [2,3].

BEFV is a negative ssRNA genome and viral particles have a bullet-like appearance or tapered shape. In addition, the virus bears spikes on the surface of envelope proteins. Five structural proteins of BEFV have been described, which comprise nucleoprotein (N), surface glycoprotein (G), large RNA-dependent RNA polymerase (L), polymerase associate protein (P) and matrix protein (M) [4-7]. Monoclonal antibody (MAb) studies of the prototype virus indicate that the G protein is the main protective antigen [8]. Four distinct antigenic sites (G_1_, G_2_, G_3_ and G_4_) on the surface of the G protein have been identified [8-10]. Antigenic site G_1_ is linear, and G_2_ and G_3_ are conformational. G_1_ reacts only with anti-BEFV antibodies, but the other antigenic sites show cross-reactivity with sera against other related viruses [11]. A blocking enzyme-linked immunoabsorbent assay (ELISA) and two indirect ELISAs for the detection of the antibodies against the G_1_ site of BEFV have been established [12-14].

BEF was first documented in China in 1955 [15]. The first BEFV strain JB76H was isolated from infected dairy cattle during the 1976 epidemic in mainland China [16]. The disease was reported to be prevalent in twenty-five provinces in mainland China from 1952 to 1991 [11,15]. From 1991 to date, because BEF is not carried out, there is a lack of detailed epidemiological data on the disease in mainland China, except for Henan Province in central China. The major BEF epidemics are shown in Table 1. The data were mainly obtained from the previous reports [11,15], while the data relating to the BEF epidemics in Henan Province from 1983 to 2011 were obtained from our monitoring system.

**Table 1:** The BEF epidemics that have occurred in mainland China (mainly from 1955 to 1991)

**Provinces**	**Years**
Guangdong	1955, 1962, 1966, 1971, 1972, 1976, 1977, 1978, 1979, 1983, 1985, 1987, 1988, 1991
Guangxi	1976, 1982
Hunan	1955, 1963, 1966, 1978, 1979, 1983–1984, 1987, 1988, 1991
Hubei	1959, 1964, 1970, 1976, 1983, 1987, 1991
Hainan	1957-1959, 1967–1969, 1971–1972, 1975–1984, 1985–1989, 1991
Henan	1949-1982, 1983, 1985, 1989, 1991, 1997, 2004, 2005, 2011
Jiangsu	1954-1955, 1966, 1976–1977, 1991
Zhejiang	1955, 1958, 1965, 1971, 1983, 1987, 1988, 1991, 2002
Fujian	1954, 1955, 1958, 1963, 1966, 1972, 1975
Jiangxi	1949-1989, 1991
Anhui	1954, 1955, 1958, 1966, 1970, 1976, 1983, 1987, 1988, 1991
Shandong	1954, 1955, 1959, 1965, 1966, 1970, 1971, 1976, 1983, 1987, 1991
Shanghai	1952, 1955, 1958, 1971, 1976, 1983, 1991
Yunnan	1965, 1973, 1975, 1977, 1978, 1982, 1983, 1986–1989, 1991
Guizhou	1955, 1957, 1969, 1976, 1983
Sichuan	1954, 1957, 1962, 1967, 1969, 1976, 1980, 1983, 1986, 1987, 1989
Xizang	1976-1979, 1985-1989
Shǎnxi	1953-1960, 1961, 1962, 1966, 1968, 1969, 1975–1978, 1982–1986, 1989, 1991
Gansu	1956, 1964, 1969, 1975, 1977, 1981, 1986, 1987, 1989
Ningxia	1957-1989
Shānxi	1954, 1959, 1991
Beijing	1956, 1966, 1976
Neimenggu	1966, 1971, 1974, 1975, 1976
Liaoning	1985, 1991
Jilin	1983, 1991

Currently, no information is available on the variation in antigenic properties and nucleotide sequences of the G gene of BEFV isolated in mainland China. In this study, the complete G genes of four BEFV strains (LS11, LYC11, JT02L and JB76H) obtained from mainland China were amplified and sequenced. For the first time, the phylogenetic relationships and antigenic variation of the G genes of BEFV, isolated from mainland China, Taiwan, Japan, Turkey, Israel and Australia, were analyzed.

## Materials and methods

### Virus isolation and identification

The JB76H strain was used in mainland China as the vaccine against BEFV. The BEFV strain JT02L was obtained from an outbreak that occurred in 2002 in Zhejiang Province. The LS11 and LYC11 strains of BEFV were isolated from blood samples of the infected dairy cattle during the 2011 epidemic in Luoyang city, Henan Province. The blood samples were collected in order to monitor BEF, which is required by the foundation item supporting for this work.

Isolation of BEFV was carried out in the brains of suckling mice and baby hamster kidney (BHK-21) cells as described previously [17]. Briefly, the blood collected from infected dairy cattle was mixed with Alsever's solution, and BEFV was concentrated decuple by centrifugation of the blood sample. Subsequently, the BEFV samples were inoculated into the brains of suckling mice and subjected to seven blind passages in suckling mice. Thereafter, BHK-21 cells were inoculated with BEFV extracted from the brains of the 7th passage infected suckling mice. The virus underwent five to ten passages in BHK-21 cells, until cytopathogenic effects (CPE) were observed.

The presence of BEFV was confirmed by reverse-transcription polymerase chain reaction (RT-PCR) as reported previously [18]. The BEFV RNAs were extracted from the infected blood and BHK-21 cells using a QIAamp viral RNA mini kit (Qiagen, Hilden, Germany). For RT-PCR, the primers were 420F (5' AGA GCT TGG TGT GAA TAC 3') and 420R (5' CCA ACC TAC AAC AGC AGA TA 3'). The forward primer 420F was used to reverse-transcribe BEFV RNA to cDNA. Subsequently, a partial fragment of the BEFV G gene was amplified using the primers 420F and 420B. After the initial denaturation at 94°C for 5min, the amplification proceeded through a total of 35 cycles consisting of denaturation at 94°C for 40s, annealing at 46°C for 1min, primer extension at 72°C for 40s and a final extension for 10min at 72°C. The expected DNA fragments were 420 base pairs (bp) in length.

### Amplification and sequencing of the BEFV G gene

The complete BEFV G gene was amplified as described in a previous report [19]. The primers were GF (5' ATG TTC AAG GTC CTC ATA ATT ACC 3') and GR (5' TAA TGA TCA AAG AAC CTA TCA TCA C 3'). The amplification procedures were carried out according to the report [19], except for the extension at 68°C for 2 min and the final extension at 68°C for 10 min.

The amplified fragments of the BEFV G gene were purified with an agarose gel DNA purification kit (TaKaRa, Dalian, China) and ligated with the pGEM-T Easy vector. Subsequently, the ligated mixtures were transformed into *Escherichia coli* DH5α. The plasmids were extracted from positive clones and then sequenced by TaKaRa (Dalian, China). The sequences obtained were deposited in the NCBI GenBank database.

### Phylogenetic analysis of the BEFV G gene sequences

The nucleotide length of the region encoding the entire ectodomain of BEFV G protein is 1527 bp [10]. An alignment of BEFV sequences corresponding to the ectodomain region were carried out using the Clustal W program [20]. The BEFV strains were isolated from mainland China, Taiwan, Japan, Turkey, Israel and Australia (Table 2). The nucleotide and deduced amino acid (aa) sequence homologies among the isolates were analyzed using the MegAlign program of DNAstar. The phylogenetic tree based on the nucleotide sequence (1527 bp) of the analyzed G genes was constructed by the neighbor-jointing method [21] with the Kimura two-parameter model [22]. The reliability of the branching orders was evaluated by the bootstrap test with 1000 replicates [23]. Phylogenetic analyses were conducted using MEGA 5 software [24]. If the nucleotide sequences of several BEFV strains had 100% homology, a representative isolate was used to construct the phylogenetic tree.

**Table 2:** Characteristics of BEFV strains used in this study

**Strain**	**Source**	**Year collected**	**Geographical origin**	**Cluster**	**Accession No.**
JB76H	Bovine blood	1976	Beijing, Mainland China	I	JQ728557
JT02L	Bovine blood	2002	Zhejiang, Mainland China	I	JQ728558
LS11	Bovine blood	2011	Henan, Mainland China	I	JQ728559
LYC11	Bovine blood	2011	Henan, Mainland China	I	JQ728560
YHL	Bovine blood	1966	Yamaguchi, Japan	I	AB462028
Hirado-6	Bovine plasma	1988	Nagasaki, Japan	I	AB462029
^a^ Hirado-9	Bovine plasma	1988	Nagasaki, Japan	I	AB462030
Amakusa-1	Bovine blood	1988	Kumamoto, Japan	I	AB462031
* ^a^ Amakusa-2	Bovine blood	1988	Kumamoto, Japan	I	AB462032
^b^ Azuma	Bovine erythrocyte	1988	Kagoshima, Japan	I	AB462033
* ^b^ ON-BEF-88-1	Bovine white blood cells	1988	Okinawa, Japan	I	AB462034
ON-BEF-88-3	Bovine white blood cells	1988	Okinawa, Japan	I	AB462035
ON-BEF-88-4	Bovine white blood cells	1988	Okinawa, Japan	I	AB462036
ON-BEF-89-1	Bovine white blood cells	1989	Okinawa, Japan	I	AB462037
ON-BEF-89-2	Bovine white blood cells	1989	Okinawa, Japan	I	AB462038
ON-BEF-89-3	Bovine white blood cells	1989	Okinawa, Japan	I	AB462039
* ^b^ Onna3	Bovine erythrocyte	1989	Okinawa, Japan	I	AB462040
ON-BEF-01-1	Bovine white blood cells	2001	Okinawa, Japan	I	AB462041
^c^ ON-BEF-01-2	Bovine erythrocyte	2001	Okinawa, Japan	I	AB462042
ON-BEF-01-3	Bovine erythrocyte	2001	Okinawa, Japan	I	AB462043
ON-04-1	Bovine blood	2004	Okinawa, Japan	I	AB462044
CS1180	Bovine blood	1982	Queensland, Australia	III	AF058321
CS1647	*Culicoides brevitarsis*	1984	Queensland, Australia	III	AF058322
CS1619			Australia	III	AF058323
CS42	*Anopheles bancrofti*	1975	Northern Territory, Australia	III	AF058324
CS1818	Bovine blood	1970	Queensland, Australia	III	AF058325
^d^ BB7721	Bovine blood	1968	Queensland, Australia	III	AF234533
* ^d^			Australia	III	NC002526
1984/TW/TN1	Bovine blood	1984	Taiwan	I	AY935239
1996/TW/TN1	Bovine blood	1996	Taiwan	I	AY935240
TN88128	Bovine blood	1999	Taiwan	I	AF208840
^e^ 2001/TW/TN1	Bovine blood	2001	Taiwan	I	AY935241
* ^c^ 2001/TW/TN2	Bovine blood	2001	Taiwan	I	AY954451
2001/TW/TN3	Bovine blood	2001	Taiwan	I	AY954452
* ^e^ 2001/TW/TN4	Bovine blood	2001	Taiwan	I	AY954453
* ^e^ 2001/TW/TN5	Bovine blood	2001	Taiwan	I	AY954454
* ^e^ 2001/TW/TN6	Bovine blood	2001	Taiwan	I	AY954455
* ^c^ 2001/TW/TN7	Bovine blood	2001	Taiwan	I	AY954456
2001/TW/TN8	Bovine blood	2001	Taiwan	I	AY954457
2001/TW/TN9	Bovine blood	2001	Taiwan	I	AY954458
2001/TW/TN10	Bovine blood	2001	Taiwan	I	AY954459
* ^e^ 2001/TW/TN11	Bovine blood	2001	Taiwan	I	AY954460
TN-2004-124	Bovine blood	2004	Taiwan	I	AY818194
2008/TR/CP62	Bovine blood	2008	Turkey	II	GQ229451
2008/TR/CP77	Bovine blood	2008	Turkey	II	GQ229452
ISR00	Bovine blood	2000	Israel	II	JN833630
ISR01	Bovine blood	2001	Israel	II	JN833631
ISR04	Bovine blood	2004	Israel	II	JN833632
ISR10/1	Bovine blood	2010	Israel	II	JN833633
ISR10/2	Bovine blood	2010	Israel	II	JN833634
ISR10/3	Bovine blood	2010	Israel	II	JN833635

Note: For the superscripts a, b, c, d and e, the same letter indicates that the nucleotide sequences of the isolates used in this report have 100% homology. The symbol * represents the BEFV isolates that were not used to produce Figures 1 and 2.

### Amino acid sequence variation of the antigenic sites of BEFV G protein

The aa sequences corresponding to the antigenic sites G_1_, G_2_ and G_3_ have been determined previously [8,10]. The sites G_1_ and G_2_ are located at the residues 487–503 and 168–189, respectively. The conformational site G_3_ is located at residues 49–63, 215–231 and 262–271. The aa sequences deduced from BEFV G genes were aligned, and the variations in the aa corresponding to the sites G_1_, G_2_ and G_3_ were analyzed. The representative BEFV strains used were isolated at different times or from different countries and areas.

## Results

### Virus isolation and identification

The DNA fragments of 420 bp were amplified from blood samples of the infected dairy cattle by RT-PCR. It was confirmed by sequence analysis that the gene fragments represented part of the BEFV G gene indicating that the disease shown in the cattle was in fact BEF.

From the outbreak of BEF in Luoyang in 2011, infected blood was collected from dairy cattle in the Songxian and Yichuanxian areas, and two BEFV strains, designated as LS11 and LYC11, were isolated by intracerebral inoculation of suckling mice and in BHK-21 cells. The infected suckling mice showed paralysis and stiffness in their hind legs on the second to third day after inoculation and died during 12–24 hours post-morbidity. The infected BHK-21 cells showed specific CPE. The specific DNA fragments of 420 bp were also amplified from the LS11 and LYC11 strains.

### Amplification and sequencing of the BEFV G gene

Complete BEFV G genes (1872 bp) were successfully amplified and sequenced from the JB76H, JT02L, LS11 and LYC11 strains. The G gene sequences of LS11, LYC11, JT02L and JB76H isolates have been assigned the accession numbers JX564637, JX564638, JX564639 and JX564640, respectively, in the GenBank database.

### Phylogenetic analysis of the BEFV G gene sequences

**Figure 1:**
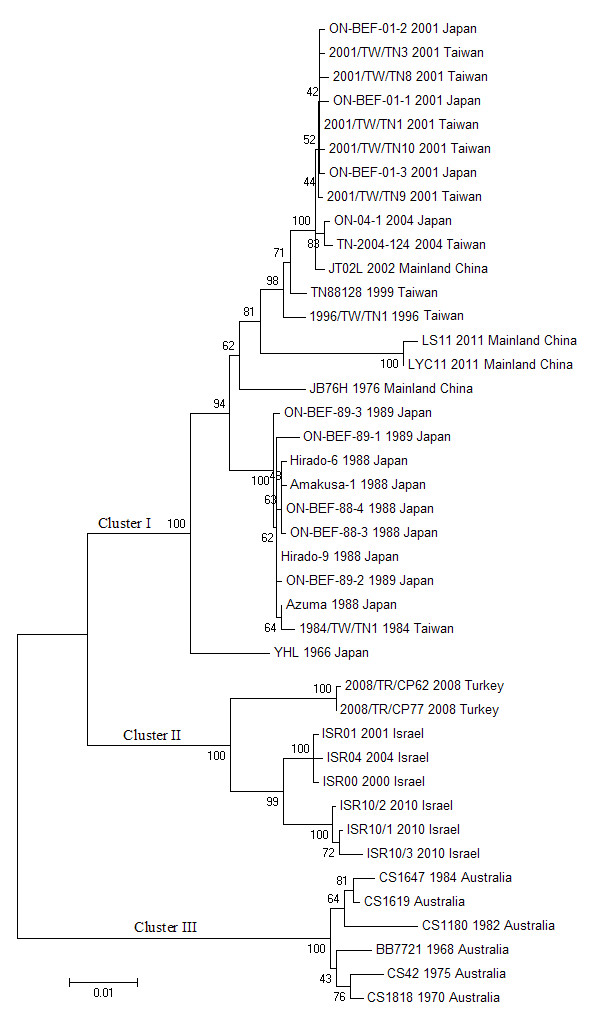
**Phylogenetic profiles of the BEFV isolates based on the comparison of the G gene sequences.** The source, year of isolation and geographical origin of each strain are indicated in the tree. The scale represents 1% sequence divergence. A total of forty-one BEFV isolates were used to construct the phylogenetic tree.

**Figure 2:**
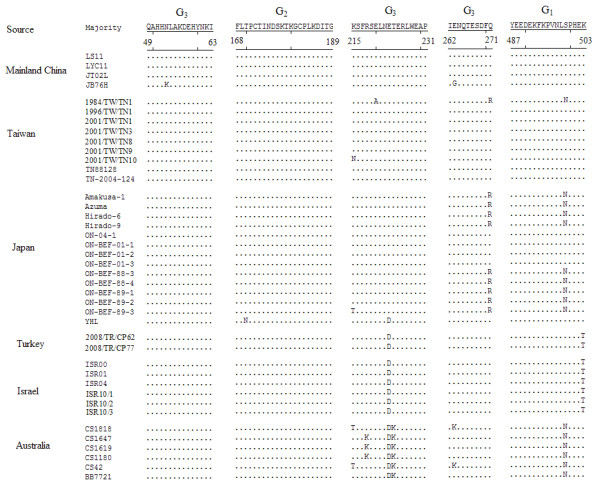
**Alignment of the aa sequences corresponding to the antigenic sites of G**_**1**_** G**_**2**_** and G**_**3**_** of BEFV G protein.** The residues differing from the sequences of the majority are denoted.

The G gene sequences of the other forty-seven BEFV isolates were obtained from the GenBank database. A total of fifty-one BEFV isolates were used in this study (Table 2), and forty-one representative strains were used to produce Figures 1 and 2.

All nucleotide and deduced aa sequences corresponding to the ectodomain region of BEFV G protein were highly conserved among the BEFV isolates obtained from mainland China, Taiwan, Japan, Turkey, Israel and Australia. The identities of the nucleotide sequences were between 89.3% and 99.9%, and those of the aa sequences were between 94.5% and 100%.

Forty-one BEFV isolates were grouped into three distinct lineages (Figure 1). Cluster I contained the strains isolated from mainland China, Taiwan and Japan. The Turkish and Israeli isolates were grouped into cluster II, and Australian strains were placed in the independent cluster III.

### Amino acid sequence variation of the antigenic sites of BEFV G protein

As shown in Figure 2, the antigenic sites of G_1_, G_2_ and G_3_ were highly conserved among the BEFV isolates obtained from mainland China and Taiwan, except for six aa substitutions in the JB76H, 1984/TW/TN1 and 2001/TW/TN10 strains. In the G_3_ site of the JB76H strain, two residues at positions 53 and 263 were substituted from N to K and from E to G respectively. Three substitutions, at positions 220 (E to A), 271 (Q to R) and 499 (S to N), were found in the 1984/TW/TN1 strain. There was a residue change at position 215 (K to N) in the 2001/TW/TN10 isolate. The residues Q at 271 and S at 499 were substituted by R and N in the Japanese strains, except for the ON-04-1, ON-BEF-01-1, ON-BEF-01-2, ON-BEF-01-3 and YHL isolates. Two substitutions at positions 170 (T to N) and 223 (E to D) were detected in the YHL strain. An additional substitution was observed at position 215 (K to T) in the ON-BEF-89-3 isolate. The three antigenic sites were completely conserved in the strains isolated from Turkey and Israel. Among the eight isolates, only two amino acids at positions 223 and 503 were substituted from E to D and from K to T, respectively. In the antigenic site G_1_ of Australian isolates, the residue was N at position 499, differing from the majority of sequences, which contained S. The substitutions were found at positions 223–224 (ET to DK) in the G_3_ site of the six Australian isolates. An additional substitution was observed at position 218 (R to K) in the CS1647, CS1619 and CS1180 isolates. Two additional aa changes were found at positions 215 (K to T) and 263 (E to K) in the CS1818 and CS42 isolates.

## Discussion

The clinical signs, morbidity and mortality associated with current cases of BEF are different from those of BEF cases reported before 2000. The current disease cases showed more severe symptoms, and the morbidity and mortality have increased significantly. Luoyang city, in Henan Province, central China, is an epidemic area for BEF, and there have been eight BEF epidemics in the area from 1983 to 2011. The three BEF epizootics in 2011, 2005 and 2004 caused considerable economic loss to dairy cattle farming. During the latest an outbreak, which occurred in 2011, the infected dairy cattle showed a sudden onset of fever, stiffness, and nasal and ocular discharges. Moreover, difficulty in breathing and shortness of breath were the most obvious clinical symptoms shown by the infected dairy cattle. Some of the severe cases died between 6 and 12 hours after infection. The morbidity was about thirty percent, and the mortality rate was about five percent. However, the morbidity was from ten to twenty percent and the mortality was lower than one percent before 2000. During the 2004 BEF epizootic, about 12,200 dairy cattle were affected, of which, 2,198 cases died. Similar data were obtained in the 2005 epizootic. In the 2011 outbreak, the infected and dead dairy cattle rose to about 32,051 and 5,360, respectively. Certainly, the numbers of dairy cattle were increased from 44,000 in 2004 and 2005 to 107,300 in 2011 (http://henan.people.com.cn/news/2011/08/02/558227.html). The high feeding density of dairy cattle and the suffocation caused by the BEF may be the leading reasons for the high mortalities.

The phylogenetic relationships of the G gene sequence of BEFV isolated in Japan, Taiwan, Turkey, Israel and Australia had been analyzed previously [25,26]. To date, the genetic relationships of BEFV derived from mainland China and those from other countries or areas have not been studied. In order to clarify the variation in the BEFV G gene with time and location, the G genes of four BEFV strains (LS11, LYC11, JT02L and JB76H) isolated from mainland China were amplified and sequenced. The G gene sequences of the three field isolates was repeatedly amplified and sequenced from infected blood, suckling brain and BHK-21 cells. The results showed that no change was found in the nucleotide sequences, indicating that the adaptation to suckling mice and BHK-21 cells through low passages had no significant effect on the nucleotide sequences of the BEFV G gene. However, it is worth noting that only one G gene sequence of the JB76H strain was used, because the original samples could not be obtained. It was unclear whether the extensive passages in BHK-21 cells affected variation in the G gene sequence of JB76H isolate.

The nucleotide and deduced aa sequences of the region encoding the ectodomain of BEFV G protein were well conserved among the BEFV isolates. In particular, the strains that originated from mainland China, Taiwan and Japan had higher identities. The corresponding sequences of the isolates derived from Turkey and Israel were highly conserved. However, the identities of the sequences were slightly lower among Australian isolates and other strains.

The phylogenetic relationships of the sequences of the ectodomain region of the BEFV G gene were analyzed in this work. The analysis revealed that the clusters of the BEFV isolates were closely related with geographical location. The strains derived from oriental areas (mainland China, Taiwan and Japan) had a close relationship. Turkish and Israeli isolates were grouped into one cluster, which had a close relationship. The Australian isolates were grouped into an independent cluster, and had a distant relationship with the Asian strains. The results revealed that the phylogenetic relationships among the BEFV isolates were closely interrelated with geographical location. Close genetic relationships among BEFV strains can be deduced if the isolates originate from adjacent areas. Similarly, the BEFV isolates derived from widely separated regions have distant genetic relationships. This may indicate that BEFV circulates in neighboring region for a long time.

The clusters of the isolates were also chronologically related. In cluster I, the JT02L strain clustered with other East Asian isolates from 2001**–**2004, suggesting that the same BEF outbreak spread through mainland China, Taiwan and Japan across the borders. The LS11 and LYC11 strains slightly diverged from the isolates from previous epizootics in East Asia, which indicated that the new BEFV possibly invaded mainland China from a neighboring area via infected vectors carried on the seasonal wind over a long distance or the import of live cattle. In fact, some evidence has shown that both winds and animal transport have an important role in trans-boundary transmission of BEFV [25,26]. The oldest Chinese mainland vaccine strain, JB76H, and the oldest Japanese strain, YHL, sat separately. The Japanese and Taiwanese isolates from 1984**–**1989 clustered together. Similar results were obtained in the clusters II and III.

The variation in the aa sequences of the antigenic sites G_1_, G_2_ and G_3_ of the BEFV isolates was analyzed. The mentioned aa sequences of the three field strains obtained from mainland China corresponded identically with those of the Japanese isolates from 2001**–**2004 and the Taiwanese strains except for the 1984/TW/TN1 and 2001/TW/TN10. The other Japanese isolates from 1988**–**1989 had the same aa sequences mentioned above except for a substitution in ON-BEF-89-3 strain. No residues were changed among the isolates derived from Turkey and Israel. Three to five substitutions were found in the antigenic sites of G_1_ and G_3_ of Australian isolates compared with the residues of the Chinese mainland strains. These results indicated that the antigenic sites G_1_, G_2_ and G_3_ of BEFV isolates that related closely in place or time were highly conserved.

## Conclusions

The sequences of the ectodomain region of the BEFV G gene were analyzed. The BEFV strains were isolated from mainland China, Taiwan, Japan, Turkey, Israel and Australia. The nucleotide and deduced aa sequences were well conserved among the isolates. A phylogenetic tree based on the nucleotide sequences was constructed, and the isolates were grouped into three clusters. The variations in the aa sequences of the antigenic sites G_1_, G_2_ and G_3_ of BEFV G protein were analyzed. The results showed that the phylogenetic relationships of the isolates were closely related to their geographical and chronological sources.

## Abbreviations

BEFV: Bovine ephemeral fever virus; BEF: Bovine ephemeral fever; G: Glycoprotein; NCBI: National center for biotechnology information; ELISA: Enzyme-linked immunoabsorbent assay; RT-PCR: Reverse-transcription polymerase chain reaction.

## Competing interests

The authors declare that they have no competing interests.

## Authors' contributions

FZ carried out the studies and drafted the manuscript. CQ gave the polish to the language of the manuscript. The two authors read and approved the final paper.
